# Primary gastric cancer presenting with a metastatic embolus in the common carotid artery: a case report

**DOI:** 10.1186/1477-7819-10-229

**Published:** 2012-10-30

**Authors:** Ying Zhang, Xiu-feng Zhang, Wei-hui Shentu, Guan-gen Yang, Zhong Shen, Pin-tong Huang

**Affiliations:** 1 Department of Ultrasound, The Second Affiliated Hospital, Zhejiang University College of Medicine, No.88 Jiefang Road, Hangzhou, Zhejiang, 310009, China; 2 Department of Colorectal Surgery, The Third People’s Hospital of Hangzhou, No. 38 Westlake Avenue, Hangzhou, Zhejiang, 310009, China

**Keywords:** Tumor embolus, Common carotid artery, Metastasis, Gastric cancer

## Abstract

Although about 30% of gastric cancers have distant metastasis at the time of initial diagnosis, metastatic tumor embolus in the main blood vessels is not common, especially in the main artery. The report presents, for the first time, an extremely rare clinical case of a metastatic embolus in the common carotid artery (CCA) from primary gastric cancer. Metastatic embolus from the primary tumor should be considered when patients present with gastric cancer accompanied by intravascular emboli. The patient should be actively examined further so as to allow early detection and treatment.

## Background

Metastatic tumor embolus in the common carotid artery from primary gastric cancer is an exceedingly rare event. Here, we report on the case of a 69-year-old male with primary gastric cancer presenting with a metastatic tumor embolus in the common carotid artery who underwent a palliative subtotal gastrectomy and endarterectomy, which is the first report of metastatic embolus in the common carotid artery in English and non-English literature.

## Case presentation

**Figure 1:**
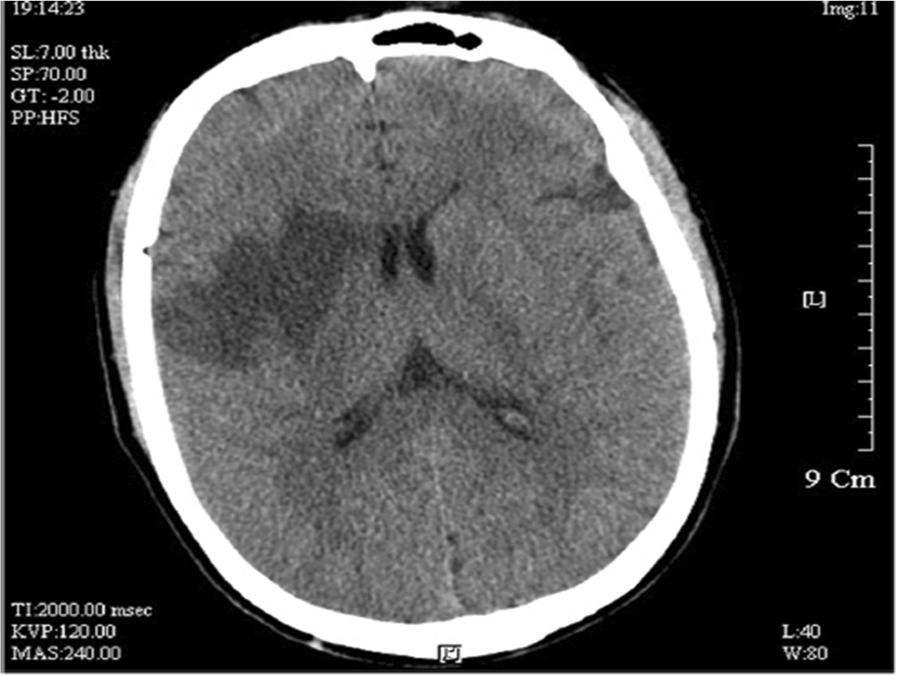
CT showed hypodense lesions in the right basal ganglia.

**Figure 2:**
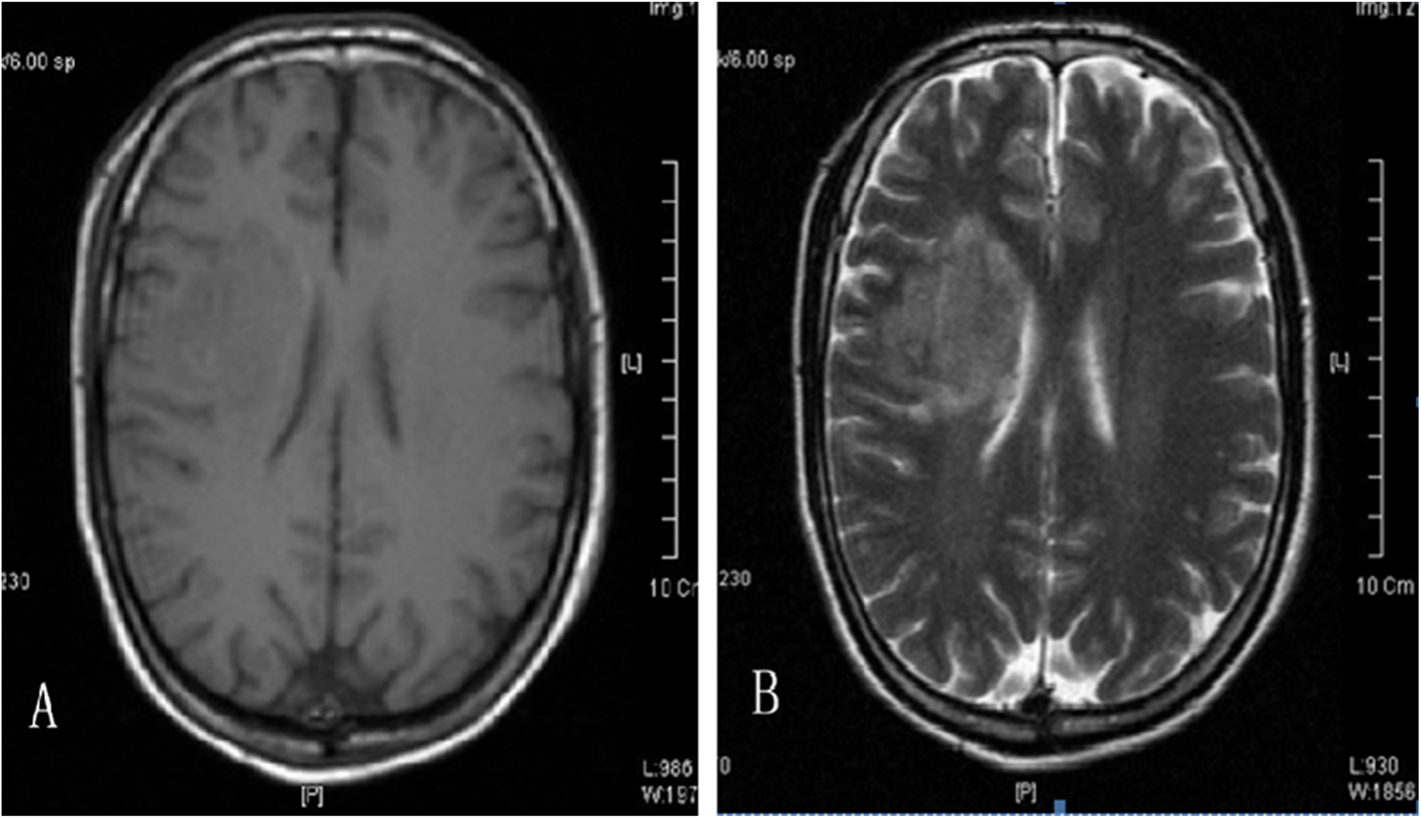
**A. MRI (T1-weighted image) showed right basal ganglia cerebral ischemic lesions. B.** MRI (T2-weighted image) showed right basal ganglia cerebral ischemic lesions.

The patient was a 69-year-old male patient who had been hospitalized for four days with sudden left limb weakness. Four days previously, during outdoor activities, this patient had felt sudden left limb weakness accompanied by instability in holding materials with his left arm, unsteady gait of his left leg and impaired speech articulation. Physical examination revealed: conscious, heart and lung (−); neurological symptoms: isocoria, light reflex was normal, mouth slightly favored the right, the left nasolabial fold was a little shallow, tongue lolled to the left side, left side paresis test (+), Pakistan’s sign of the left side (±), Pakistan’s sign on the right side (−), limb muscle strength and muscular tension was normal. Emergency cranial computed tomography (CT) scan in another hospital showed that hypodense lesions in the right basal ganglia were detected and the diagnosis of cerebral infarction was considered (Figure 1). In our hospital, magnetic resonance imaging (MRI) examination revealed right basal ganglia cerebral infarction and ischemic lesions (Figures 2-A.B). Carbohydrate antigen (CA) 19–9: 12.35U/ml, carcinoembrionic antigen (CEA): 25.28ng/ml, CA72-4: 21.73U/ml. The patient denied having ‘hepatitis, tuberculosis, diabetes, hypertension, coronary heart disease’ and gave a family genetic history. He related a history of ‘chronic gastritis’ for the last three years, and having occasional upper abdominal discomfort after meals for the last six months. His brothers and sisters also had history of gastritis.

**Figure 3:**
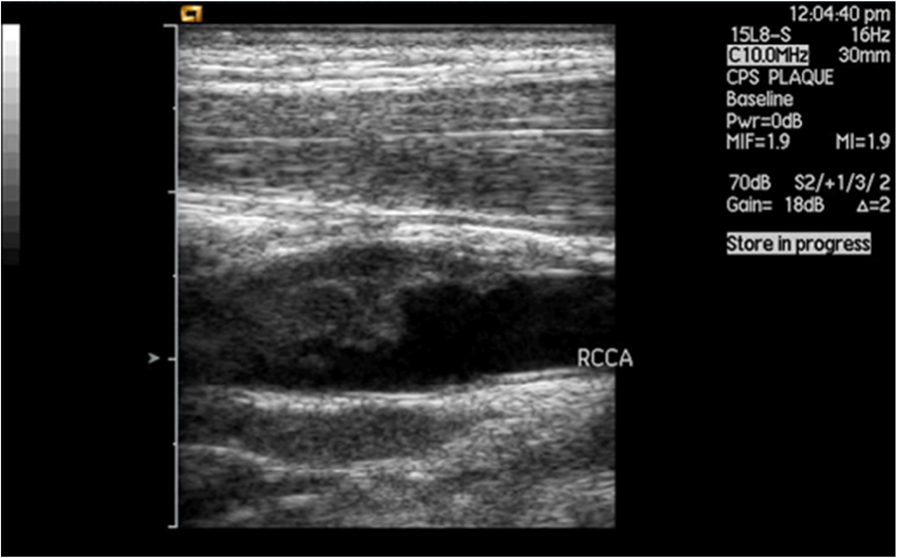
**Carotid ultrasonography showed that the diameters of bilateral common carotid artery were normal.** A hypoechoic mass with irregular contour was observed at the right common carotid artery. The size of the mass was about 29 × 7mm, with heteroechogenicity.

Conventional carotid ultrasonography showed that the diameters of the bilateral common carotid artery were normal. The intima-media thickness (IMT) of the left side was 0.8 mm. A hypoechoic mass with an irregular contour was observed at the right common carotid artery. The size of the mass was about 29 × 7 mm, with heteroechogenicity. A pedicled structure, which connected the mass with the common carotid artery wall, could be seen and the mass could swing slightly following carotid pulsation (Figure 3).

**Figure 4:**
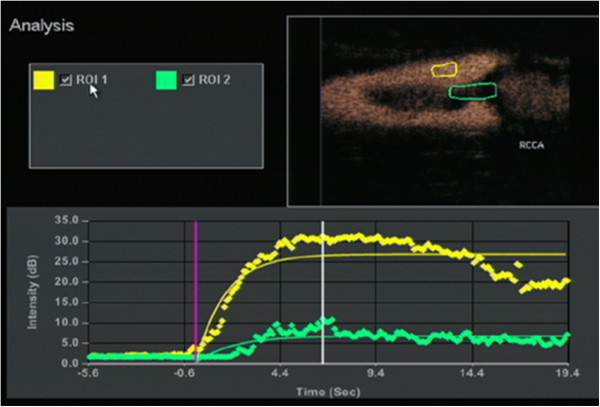
Time-intensity curve analysis in two user-defined regions of interest within the common carotid artery (yellow curves) and the lumen of the hypodensity lesions (green curves).

The contrast-enhanced ultrasonography (CEUS) was then performed and displayed the mass located on the posterior common carotid artery wall. About 14 seconds after the injecting of the contrast agent, the carotid arterial lumen enhanced first. About 18 seconds after the injecting of the contrast agent, the visible point-like enhancement within the hypoechoic mass could be seen. At approximately 32 seconds, the enhancement of the mass reached the peak, and began to wash out at about 48 seconds (Figure 4).

Considering the ‘three-year history of chronic gastritis’, an abdominal ultrasound examination was routinely performed and revealed a thickened gastric antrum wall. Further double contrast-enhanced ultrasonography (DCEUS) showed that the wall of the gastric angle and antrum were obviously thickened with hypoechoic inner echogenicity. Five layers of normal gastric wall had disappeared; the basal boundary was obscure. The hypoechoic lesion involved all layers of the gastric wall. Enlarged intra-abdominal lymph nodes with clear borders were detected around the gastric pylorus. The size of the largest lymph node was about 10 × 8 mm.

**Figure 5:**
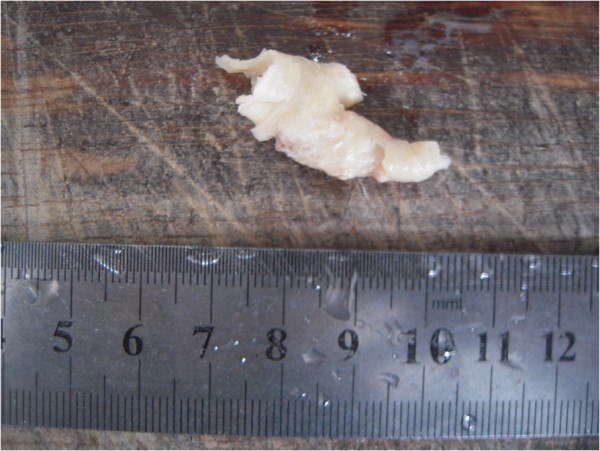
The gross specimen of metastatic tumor embolus in the common carotid artery after endarterectomy.

**Figure 6:**
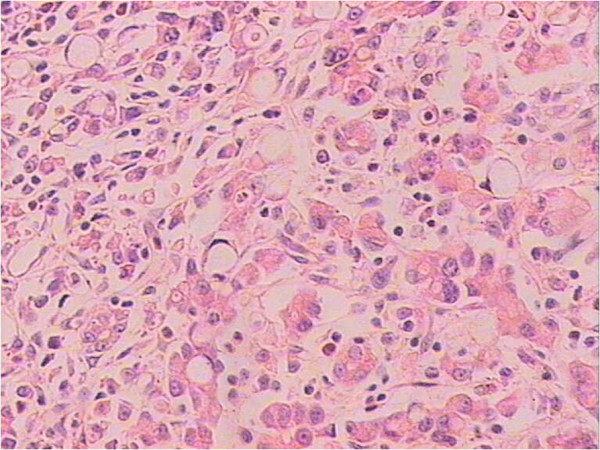
Histological findings with H&E staining of embolus in the common carotid artery showed the poorly differentiated adenocarcinoma and partial signet-ring cell carcinoma (×400).

The preliminary impression from the ultrasound was advanced gastric cancer with carotid arterial tumor embolus, which was confirmed by the biopsy from the gastroscope and specimens obtained from the palliative, subtotal gastrectomy and endarterectomy. The poorly differentiated adenocarcinoma and partial signet-ring cell carcinoma in the gastric antrum was confirmed by histopathological examination (Figures 5, 6).

## Discussion

The routine carotid ultrasonography was performed because of the occurrence of the cerebral embolism. The tumor embolus in the common carotid artery was subsequently found and showed a hypoechoic mass with an uneven surface at the right common carotid artery bifurcation. It was different from the common carotid atherosclerotic plaque. The imaging features of carotid plaque in ultrasound examinations have been reported in many published works [1-3]. Considering the history, a middle-aged patient, with no obvious thickening of the common carotid artery intima, without hypertension, diabetes, coronary heart disease and other risk factors for cerebrovascular disease, and the characteristics of the carotid ultrasonography: a hypoechoic mass with a pedicle, the typical ‘fast-in, fast-out’ pattern which is not consistent with a thrombus and atherosclerotic plaque, the hypoechoic mass was suspected to be a tumor embolus. Given the complication of the history of chronic gastritis, abdominal ultrasound was ordered and found that there was nonuniform thickening of the gastric antrum wall. DCEUS has been widely used for diagnosis of gastric cancer in China [4]. It can be used in the preoperative staging of gastric cancer and has been widely applied in clinics. Many published works have reported on this method, which combines an ultrasonic oral contrast agent and a bolus injection of SonoVue to display the gastric wall. The method can help us to observe features of thickening and perfusion type of the gastric wall. The results of the DCEUS of this patient showed diffuse nonuniform thickening of the gastric wall, the lesion penetrated the serosa, there was a fast perfusion pattern, and several lymph nodes could be seen around abdominal aorta. The results of the DCEUS were consistent with the gastroscope: advanced gastric cancer. Lymph node and hematogenous metastasis (especially in the portal vein) are common pathways in advanced gastric cancer [5]. Considering the common carotid artery tumor embolus had metastasized from the advanced gastric cancer, we corrected the original diagnosis.

## Conclusion

This report presented, for the first time, an extremely rare case of a metastatic tumor embolus in the common carotid artery from primary gastric cancer. The case described was a clinical entity that can be easily misdiagnosed as atheromatous plaque, thrombus, and so on. Metastatic embolus from the primary tumor should be considered in patients with gastric cancer accompanied by intravascular emboli, and patients should be actively examined further so as to allow early detection and treatment.

## Consent

Written informed consent was obtained from the patient for publication of this case report and any accompanying images.

## Abbreviations

CCA: Common carotid artery; CEUS: Contrast-enhanced ultrasonography; CT: Computed tomography; DCEUS: Double contrast-enhanced ultrasonography; IMT: Intima-media thickness; MRI: Magnetic resonance imaging.

## Competing interests

The authors declare that they have no competing interests.

## Authors’ contributions

YZ performed the literature review, drafted and co-wrote the manuscript with XFZ. GGY and ZS revised the manuscript for intellectual content. WHST and PTH evaluated the ultrasound features and contributed to the ultrasound part of manuscript. All authors have read and approved the final version of the manuscript.

## Authors’ information

Xiu-feng Zhang was the co-first author.
